# Electrophysiological Correlates of Strategic Monitoring in Event-Based and Time-Based Prospective Memory

**DOI:** 10.1371/journal.pone.0031659

**Published:** 2012-02-21

**Authors:** Giorgia Cona, Giorgio Arcara, Vincenza Tarantino, Patrizia Silvia Bisiacchi

**Affiliations:** 1 Department of General Psychology, University of Padua, Padua, Italy; 2 Department of Information Engineering, University of Padua, Padua, Italy; 3 Department of Clinical and Experimental Medicine, Centro Interdipartimentale di Ricerca sulla Modellistica delle Alterazioni Neuropsichiche in Medicina Clinica, University of Padua, Padua, Italy; University College London, United Kingdom

## Abstract

Prospective memory (PM) is the ability to remember to accomplish an action when a particular event occurs (i.e., event-based PM), or at a specific time (i.e., time-based PM) while performing an ongoing activity. Strategic Monitoring is one of the basic cognitive functions supporting PM tasks, and involves two mechanisms: a retrieval mode, which consists of maintaining active the intention in memory; and target checking, engaged for verifying the presence of the PM cue in the environment. The present study is aimed at providing the first evidence of event-related potentials (ERPs) associated with time-based PM, and at examining differences and commonalities in the ERPs related to Strategic Monitoring mechanisms between event- and time-based PM tasks.

The addition of an event-based or a time-based PM task to an ongoing activity led to a similar sustained positive modulation of the ERPs in the ongoing trials, mainly expressed over prefrontal and frontal regions. This modulation might index the retrieval mode mechanism, similarly engaged in the two PM tasks. On the other hand, two further ERP modulations were shown specifically in an event-based PM task. An increased positivity was shown at 400–600 ms post-stimulus over occipital and parietal regions, and might be related to target checking. Moreover, an early modulation at 130–180 ms post-stimulus seems to reflect the recruitment of attentional resources for being ready to respond to the event-based PM cue. This latter modulation suggests the existence of a third mechanism specific for the event-based PM; that is, the “readiness mode”.

## Introduction

In everyday life, individuals are often required to retrieve intentions from memory for correctly fulfilling a task at the appropriate time. This ability is the result of a multicomponential process that has been named prospective memory (PM) [1]–[3]. Remembering to put fuel in the car or to take medication are just some examples of PM activities and they demonstrate how much the PM is an essential ability.

Einstein and McDaniel [4], [5] have distinguished two types of PM tasks depending on the kind of stimulus triggering the intention retrieval; namely event-based PM tasks and time-based PM tasks. In an event-based PM task, individuals have to remember to perform an intended action when a particular event, the PM cue, occurs (e.g. to put fuel in the car in the presence of a gas station). On the other hand, in a time-based PM task, individuals have to remember to perform the intended action at a specified time or after a time interval (e.g., taking medication at noon or every twelve hours). An intrinsic feature of event-based and time-based PM tasks is that individuals accomplish the intended actions while performing other ongoing activities and have to retrieve them without an explicit prompt from the environment that instigates the recollection of those actions. Several studies showed that in event-based PM tasks, Strategic Monitoring supports the intention retrieval and consists of preparatory attentional and memory processes needed for monitoring the environment for the PM cue occurrence [6], [7], [ cf. 8 for the description of the situations eliciting Strategic Monitoring]. Strategic Monitoring, however, as conceptualized in the PM models [9]–[12], cannot be completely applied to the time-based PM tasks where internal, but not external, PM cues are associated with the intended action. Specifically, according to Guynn [6], [10], Strategic Monitoring is thought to be composed of two independent mechanisms: retrieval mode and target checking. Retrieval mode is thought as a “neurocognitive task set to treat stimuli (external or internal) as cues to retrieve intentions” [6], or, in other words, as a sustained condition of readiness in order to respond to the incoming PM cues and, therefore, to appropriately execute the intention. This mechanism is based on maintaining the representation of intention active in memory. In contrast, target checking is a more intermittent mechanism. It consists either in monitoring the environmental stimuli for the detection of the PM cues - in event-based tasks - or in monitoring the passage of the time (e.g., by clock checking), in time-based tasks. Given these assumptions, event-based and time-based PM tasks are similar in recruiting a retrieval mode mechanism, since they both require the intention to be maintained active in memory. On the other side, they are mediated by different mechanisms of target checking (i.e., checking the environment for the PM events *versus* checking the clock in time-based tasks).

Nevertheless, although time-based PM tasks are supposed to engage different monitoring processes from those implied in event-based PM tasks, a few studies have directly tested this hypothesis [e.g. 13]–[16]. An index commonly used in these studies to quantify Strategic Monitoring is the PM interference effect, which is the decline of the ongoing performance (i.e., slowing of reaction times and/or decrease in accuracy) when a PM task is added [7], [17], [18]. Early works claimed that time-based PM tasks would show a greater PM interference effect than event-based tasks because they require a higher degree of self-initiated and controlled processes [14]. Later studies have pointed out that event-based PM tasks produce greater PM interference effects than time-based tasks [15], [16], [19].

Among these, the study by Tarantino and collaborators [20] has found that predictability of the PM cue is a crucial factor in determining the extent to which Strategic Monitoring processes are engaged in the event- and time-based PM tasks [see also 16]. Specifically, in event-based PM tasks, the PM cue occurrence is not beyond the control of the individuals, thus individuals carry out a continuous monitoring process in order to be ready to detect the PM cue. On the contrary, in time-based tasks, in which the PM cue (i.e., the appropriate time) is intrinsically predictable, individuals are engaged in time monitoring (i.e., target checking) only periodically, as the occurrence of the PM cue approaches. The idea of a periodically monitoring in time-based tasks is in line with the test-wait-test-exit model [21]–[23]. According to this model, the time checks (due to the rehearsals of time-based intentions) usually happen prior to the appropriate time to remember and they are periodically repeated until the successful execution of the intention.

The Strategic Monitoring mechanisms supporting PM were also objects of interest for neuroimaging studies [24]–[29], [ see 30 for a review]. Nevertheless, almost all of such studies focused on event-based PM tasks. The most used contrast to show brain activations specific to strategic monitoring was between “uncontaminated” ongoing trials performance (i.e., performance of the ongoing task alone) and the ongoing trials performance while a PM intention was maintained in memory [e.g.], [ 24]–[26], [28]–[29]. These studies converged in indicating the anterior part of the prefrontal cortex (aPFC, BA 10) as the core brain region in maintaining active the intention during the ongoing activity. Surprisingly, so far only one study has compared the neural substrates of time-based and event-based PM tasks [31]. The authors found a dissociation within the aPFC depending on the type of PM tasks: the activation of a more superior area of the aPFC in the event-based condition and a more inferior area in the time-based one. Furthermore, when a self-estimation of time was required, a more superior and closer to the midline activation of the aPFC was shown compared to the condition in which time monitoring was facilitated by the aid of a clock. The causal role of prefrontal cortex in strategic monitoring processes of event-based PM tasks was evidenced in a transcranial magnetic stimulation (TMS) study [32]. Indeed it showed that stimulation of the right dorsolateral prefrontal cortex led to an impaired ongoing performance only when a PM task was added to the ongoing task (but not when the ongoing task was executed alone).

As for neuroimaging studies, all the studies that used the event-related potentials (ERPs) technique focused only on event-based PM [33]–[40], [ see 41 for a review]. Significant modulations were found in the ERPs elicited by ongoing trials, in which the occurrence of the PM cue was monitored, and for this reason they were interpreted as reflecting the allocation of attention required for monitoring the presence of the PM cue [37], [42]. Specifically, West and collaborators highlighted a sustained activity expressed in an enhanced negativity over occipital regions coupled with a positivity over frontal regions, beginning at roughly 200–400 ms after stimulus onset [38], [39]. Another study suggested that such a sustained frontal/occipital-parietal activity might be related to target checking [39], nevertheless it did not allow to completely excluding the possibility that it also reflected the retrieval mode [40]. Indeed, a later study considered the long-lasting activity expressed over frontal and posterior regions a likely candidate to be the ERP correlate of retrieval mode [40]. Other works that have investigated the effect of Strategic Monitoring on the ERPs reported similar ERP modulations, mainly expressed over the frontal regions [34], [35]. These findings seem to confirm a recruitment of the frontal lobe in Strategic Monitoring, supporting previous neuroimaging studies [e.g. 24], [29]. A recent study [36] showed that Strategic Monitoring may influence also the earlier ERP components. Specifically, it revealed an enhanced early visual perceptual component at 140 ms post-stimulus over occipital-parietal regions. Furthermore, an enhanced occipital-parietal negativity and centro-frontal positivity were found reaching the maximum of amplitudes at 220 ms post-stimulus. This study supports the idea that the preparatory attention required for Strategic Monitoring may act by improving processing of PM cue features.

In summary, although previous studies suggest that the execution of event- and time-based PM tasks involves different mechanisms of monitoring, the neural correlates underpinning the two PM tasks have, nevertheless, been poorly compared. Moreover, it is quite surprising that no electrophysiological study has ever focused on investigating the ERP correlates of time-based PM. Therefore, our study is aimed at providing the first evidence of the brain electrical activity related to time-based PM, and at examining differences and commonalities in the ERP correlates of Strategic Monitoring between time- and event-based PM tasks. To this end, the ERPs elicited by ongoing stimuli were analysed in a baseline block, in which individuals were required to perform merely the ongoing task; and compared with those of a PM block, in which individuals were required to perform simultaneously the ongoing and the PM tasks (either event-based or time-based). Importantly, we compared the ERPs elicited by the same ongoing trials in two PM conditions: one in which individuals were required to accomplish an event-based intention, and the other in which individuals were required to accomplish a time-based intention. We hypothesized that similarities and differences in the recruitment of Strategic Monitoring mechanisms (i.e., retrieval mode and target checking) between time-based and event-based PM tasks should lead to similarities and differences in the modulations of the ERPs in ongoing trials between the two PM tasks. Specifically, the retrieval mode, conceptualised as the process of maintaining the intention continuously active in memory [6], [10] would be common between event-based and time-based PM tasks; for this reason, it should be reflected in a similar pattern of sustained ERP modulations in the two PM tasks. Furthermore, we expected this ERP activity to be more expressed over frontal regions, in line with the majority of previous neuroimaging findings [e.g. 25], [43]–[45]. On the other hand, target checking is qualitatively different between event-based and time-based tasks, and it would be engaged at different moments between the two PM tasks. In event-based tasks, target checking operates by monitoring the ongoing stimuli to detect the presence of the PM cue, therefore it would be closely linked to the occurrence of the ongoing stimuli. On the contrary, in time-based tasks, this mechanism is mediated by checking the clock; hence it does not imply that the ongoing stimuli are monitored. Since the ERPs were time-locked to the onset of the ongoing stimuli, possible modulations expressed on the ERPs in the event-based, but not in time-based task, might be associated with this additional process of verifying whether ongoing stimuli contain the PM cue, which is a process specifically required to fulfil event-based intentions.

## Materials and Methods

### Ethics Statement

The study was approved by the ethical committee of the Faculty of Psychology of the University of Padua and was conducted according to the principles expressed in the Declaration of Helsinki. All the participants were informed about the general procedure of the experiment and signed a written consent form.

### Participants

Twenty-nine students, recruited from the Faculty of Psychology at the University of Padua, took part in the study. They were randomly assigned to one of two PM conditions: fourteen students were enrolled in the event-based PM condition, and fifteen students in the time-based PM condition. Participants in the event-based PM condition had a mean age of 23.71 years (SD = 3.31; range = 20–34; 12 females); participants in the time-based PM condition had a mean age of 23.81 years (SD = 2.01; range = 21–28; 10 females). They were all right handed, as measured by the Edinburgh Handedness Inventory [46], with normal or corrected-to-normal vision, and without neurological or psychiatric pathologies. They either received course credits or € 25 for their participation to the study.

### Materials and procedure

Both the event-based and the time-based PM conditions consisted of two blocks. In the first block, composed of 40 trials, participants were asked to perform merely the ongoing task (*baseline* block). In the second block, which included a total of 350 trials, participants were required to perform a PM task in addition to the ongoing task (*PM* block). Similarly to previous studies [34], [35], the baseline block was administered before the PM block, in both the groups, in order to avoid a potential long-lasting interference effect of PM instruction on the baseline block. This consideration is driven by the fact that engagement of Strategic Monitoring has been still shown in tasks even though the PM intentions were no more relevant [16], [39]. In order to avoid any contamination by the PM intention, the PM instructions were given only after the baseline block. A between-subjects design was used, and therefore participants performed the event-based PM task or the time-based PM. In this way, we were sure to have a pure measure of the maintenance of a time-based or an event-based PM intention.

In both the conditions, at the beginning of the *baseline* block, ten practice trials were run to familiarize participants with the ongoing task.

### Ongoing task

The ongoing task was adapted from the dual-task paradigm used by Bisiacchi and collaborators [33], and consisted of white strings of five letters, pseudo-randomly presented at the centre of a black computer screen. The letters in the first, third and fifth positions were always identical, whereas the letters in the second and fourth positions could be same or different. Participants were instructed to press a key on a response box with their right index finger if the letters in the second and fourth positions were the same (e.g., DFDFD) and another key with their right middle finger if they were different (e.g., DFDGD). All responses were given with the right hand, and response keys were counterbalanced across participants. Each trial began with a blank screen with a pseudorandom duration (ranging from 1700 to 2600 ms). The five-letter string was then displayed either for 1600 ms or until the participant response. A second blank screen followed the string presentation. The duration of this second blank screen was online determined such as that overall duration of stimulus presentation plus the second inter-trial interval was 3000 ms. No feedbacks on performance were provided.

### Event-based PM condition

In the PM block of the event-based PM condition, in addition to the ongoing task, participants were asked to press the red key on the left side of the response box with their left index finger whenever the letter ‘B’ (PM cue) appeared on the second and/or fourth positions (e.g. FBFGF). The letter ‘B’ never occurred in the other, non-target, positions (i.e., on the first, third, or fifth position).

When the PM cue occurred, participants were asked to perform first the ongoing task (i.e., to press the key corresponding to the same/different decision) and then to press the red key to perform the PM task. The total number of PM cues across the task was five (1.43% of the trials). They took place in an unpredictable way, but roughly every five minutes in order to parallel the time-based PM condition. Participants were not informed about the frequency of the occurrence of the PM cue.

### Time-based PM condition

In the PM block of time-based PM condition, in addition to the ongoing task participants were instructed to press a key on the left side of the response box with their left index finger, every 5 min from the beginning of the task, trying to be as accurate as possible. When the key was pressed, a digital clock appeared on the centre of the screen, showing the exact time in minutes and seconds. The digits had the same font and colour as the letters of the ongoing task. Participants were not informed about the duration of the PM block, which lasted about 27 minutes. This duration allowed the participants to perform up to five PM responses (i.e., at 5:00, 10:00, 15:00, 20:00, 25:00 min), as in the event-based PM condition. To help them to estimate the passing of time, they had the opportunity to check the digital clock in any moment of the task by pressing another key (labelled with a clock icon on the response box) with their left middle finger. They were instructed to feel free to check the clock whenever they liked and as often as they needed. They were also instructed not to count the trials elapsed to estimate time. When they pressed the key to check time, the digital clock appeared in the centre of the screen.

### Electrophysiological recording and data analysis

EEG was recorded (EEG equipment: System Plus, Micromed, Mogliano Veneto, Italy) from an array of 30 Ag/Ag Cl scalp electrodes mounted on an elastic cap (ElectroCap International, Inc.) and positioned according to the 10–20 International System [47]. The montage included the following scalp positions: Fp1, Fpz, Fp2, F7, F3, Fz, F4, F8, FC3, FCZ, FC4, T3, C3, Cz, C4, FT7, FT8, T3, T8, T5, CP3, CPZ, CP4, P3, PZ, P4, T6, TP7, TP8, O1, O2 and right mastoid. Eye movements were monitored by two electrodes, with one electrode placed above the right eye, and one placed on the external *canthi* of the left eye. The EOG (electrooculogram) was recorded with a bipolar montage. All electrodes were referenced to the left mastoid and re-referenced offline to the average of the left and right mastoids. The ground electrode was placed in AFZ. Data was recorded with a band-pass filter set at DC-50 Hz and digitized at a sampling rate of 512 Hz. Electrode impedance was kept below 5 kΩ. Data processing was performed with EEGLAB 8.0.3.4b [48], running under Matlab environment (Version 7.4.0, MathWorks, Natick, MA, USA). Continuous EEG was resampled at 256 Hz and filtered between 0.1 Hz and 100 Hz. Then, it was segmented into epochs starting −3000 ms before the onset of the stimulus and ending 3000 ms post-stimulus. Epochs were locked to the presentation of ongoing stimuli (i.e., letter strings). Artifact correction was done on these epochs by using the Independent Component Analysis (ICA) toolbox in EEGLAB [48]. ICA allows identification of independent signals (i.e., independent components) in the data. Among the independent components identified by ICA analysis, it is possible to distinguish which ones are mostly related to artifactual signals, such as blinks and ocular movements [49]. In the time-based condition, epochs containing clock checks or PM responses were excluded from the analyses. Epochs were then digitally filtered with a low-pass 30 Hz filter. Afterwards, epochs were re-segmented, including 200 ms of pre-stimulus baseline and 1200 ms post-stimulus activity. Finally, epochs were averaged offline according to the block type (baseline and PM). In the event-based condition, epochs containing PM cues were excluded from the analysis. In both the conditions, only epochs with correct responses were analyzed. In addition, epoch rejection was performed with a cut-off of ±100 µV. In the event-based PM condition an average of 5.18% (SD = 4.95) in the baseline block, and an average of 5.32% of epochs (SD = 2.98) were removed from the analyses in the PM block. In the time-based PM condition an average of 1.33% of epochs (SD = 1.85) were rejected in the baseline block, and an average of 7.25% of epochs (SD = 5.20) were rejected from the PM block.

### Statistical analyses

Statistical analyses on averaged ERP data were performed using R, release 2.13.1 [50]. All ANOVAs were performed using the *ez* package [51].

The considered dependent variables were: mean accuracy and reaction times (RTs) of the ongoing and PM task, and mean ERP amplitude on correct ongoing trials.

In the time-based PM condition, a PM response was considered correct if the participant pressed the key for the PM response within ±15 sec from the target time (e.g. for the 5.00 min response, a response within 4.45 and 5.15 min was considered as accurate). Mean accuracy and RTs of the ongoing task were investigated by means of a 2×2 mixed ANOVA. The ANOVA included a between groups independent variable, Condition, with two levels (event-based, time-based); and a within group factor, Block, with two levels (baseline, PM).

In the time-based PM condition, a further 5×5 ANOVA was run to analyze the changes in frequency of time monitoring across the PM block. This ANOVA included two independent factors: PM response order (five levels: from the first to the fifth PM response) and the five minutes preceding each PM response (five levels: from one to five minutes). The first independent variable was included to investigate changes in frequency of time monitoring across the whole PM block, whereas the latter was included to investigate the changes in frequency of time monitoring as the time associated with a PM response (i.e., 5^th^ minute and so on) approaches.

**Figure 1:**
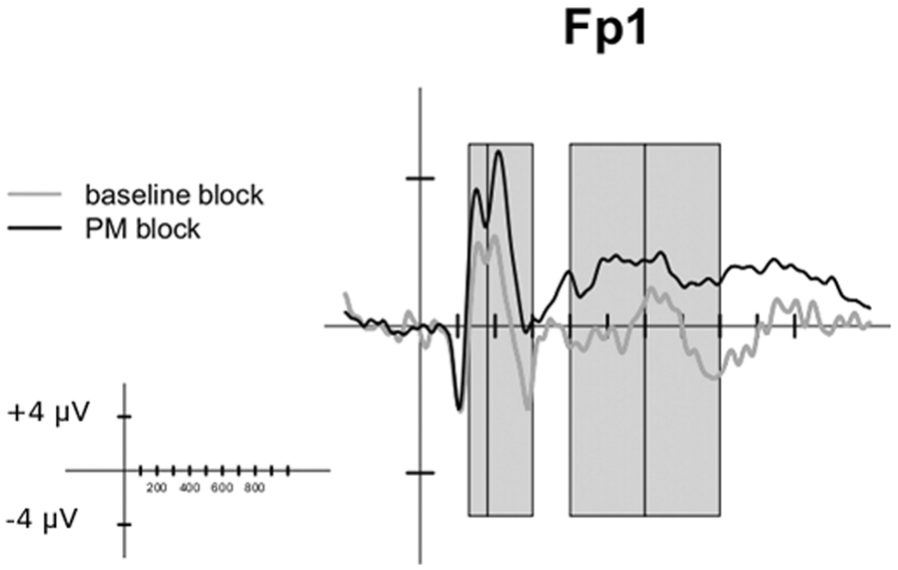
Time windows considered for statistical analysis. The plot shows the time windows considered for the statistical analysis (the ERPs shown are referred to the Time-Based condition). The four grey areas highlight the time windows considered: 130–180 ms, 180–300 ms, 400–600 ms, and 600–800 ms.

ERP analyses were conducted on four time windows on a subset of electrodes. The choice of the electrodes was driven by the previous findings in the ERP literature of PM [34]–[36] and by a visual inspection of the regions where the effects were mainly expressed (Fp1, Fp2, F3, F4, P3, P4, O1, O2). Concerning the time windows investigated (Figure 1), the first (130–180 ms) and the second (180–300 ms) time windows captured respectively the first and the second positive peaks. Two further time windows (400–600 ms, 600–800 ms) were defined to investigate the differences associated with the later modulations. Separate 2 (Condition: event-based or time-based)×2 (Block: baseline or PM)×8 (Electrodes) ANOVAs were conducted on each time window. Post-hoc contrasts were performed to explore the significant effects evidenced by ANOVAs. In all ANOVAs, Mauchly's test was used to check sphericity assumption and Greenhouse–Geisser correction for sphericity departures was applied when necessary [52]. The effect size was quantified by means of η_G_^2^ calculation [53].

## Results

### Behavioural results

Mean accuracy of the PM task was high in both the event-based and time-based PM conditions (M = 0.87, SD = 0.15, range = 0.6–1; M = 0.81, SD = 0.25, range = 0.2–1, respectively). All participants were able to recall the PM instruction in both the PM conditions.

The ANOVA on RTs of ongoing task showed no significant effects neither of Condition [F(1,27) = 0.58, p = 0.45, η_G_^2^ = 0.017], nor of Block [F(1,27) = 0.033, p = 0.86, η_G_^2^<0.001], nor of the interaction Condition×Block [F(1,27) = 0.005, p = 0.94, η_G_^2^<0.001]. Likewise to RTs, the ANOVA on the mean accuracy of the ongoing task showed no significant effects of Condition [F(1,27) = 0.06, p = 0.81, η_G_^2^ = 0.001], Block [F(1,27) = 0.80, p = 0.38, η_G_^2^<0.010], and Condition×Block [F(1,27) = 0.18, p = 0.67, η_G_^2^ = 0.002] (see Table 1).

**Table 1:** RTs and percentage of accuracy to the ongoing task.

	RT	ACC
**EVENT**		
(n = 14)		
baseline block	790.08 (108.24)	96.07 (3.88)
PM block	794.40 (101.04)	96.41 (2.65)
**TIME**		
(n = 15)		
baseline block	762.15 (128.47)	96.00 (5.07)
PM block	764.11 (110.27)	97.00 (1.69)

Mean values (and standard deviations) of RTs in milliseconds and percentage of correct responses to the ongoig task, in baseline and PM blocks for event- and time-based conditions.

Since in the present study the PM block always followed the baseline block, the PM interference effect (i.e., the decline of the ongoing performance when a PM task is added) could be masked by the speeding associated to practice effect. One possibility to disentangle these two potential effects is running a further analysis inserting the trial number as a covariate (i.e., each trial is associated with the ordinal number indicating its position within the whole experiment, i.e., the 1st trial, the 2nd trial, and so on, regardless of the block it belongs). We performed this analysis employing mixed effect regression modelling [54]. The mixed model approach is becoming increasingly used in many scientific fields [55], [56] because of its enhanced statistical power. Results of this analysis (see also File S1 for details on this analysis) showed that taking into account the practice effect, the PM interference effect was found in both the PM conditions.

For the time-based PM condition, a further analysis was conducted on the frequency of time monitoring, i.e. of clock-checks. The results of the ANOVA showed a significant effect of the minute preceding a PM response [F(1,14) = 29.75, p<0.001, η_G_^2^ = 0.65], revealing that the frequency of the clock-checks increased approaching the PM response, independently of whether the PM response was the first, the second, the third, the fourth or the fifth. Indeed, post-hoc analysis showed that clock-check frequency was higher in the fifth minute preceding a PM response compared to all the other minutes (all *ps*<0.01). Moreover, the frequency of clock-checks was higher in the fourth minute compared to the first, second and third minutes, in which the frequencies were similarly low (all *ps*<0.01).

### ERP results

**Figure 2:**
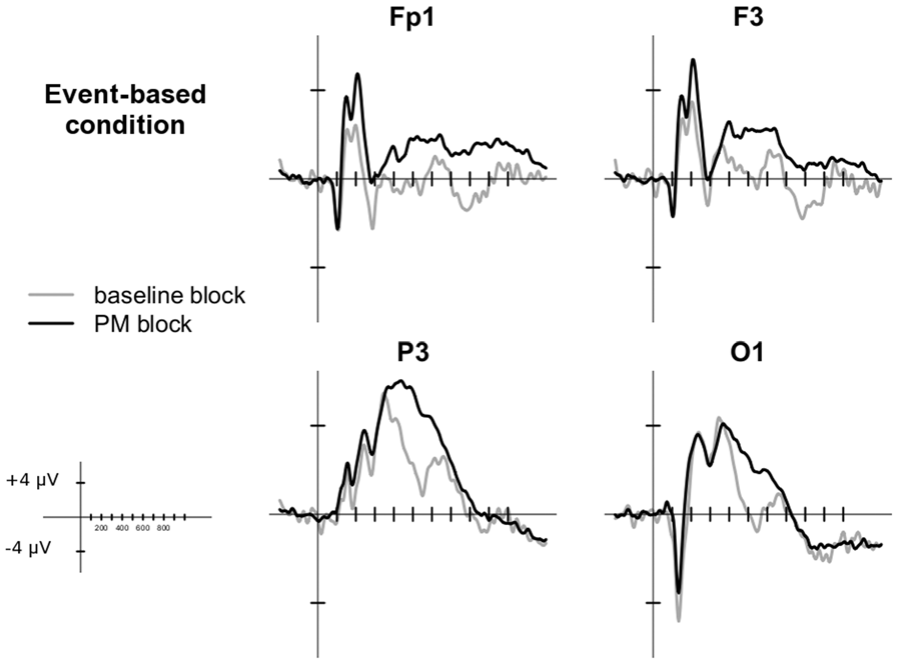
Grand average ERP waveforms for Event-based PM condition. The plots show the ERPs time-locked to ongoing trials in baseline block (gray line) and in PM block (black line) of the electrodes in which the effects were mainly expressed. Since no between-hemisphere differences were found, only left electrodes are reported here.

**Figure 3:**
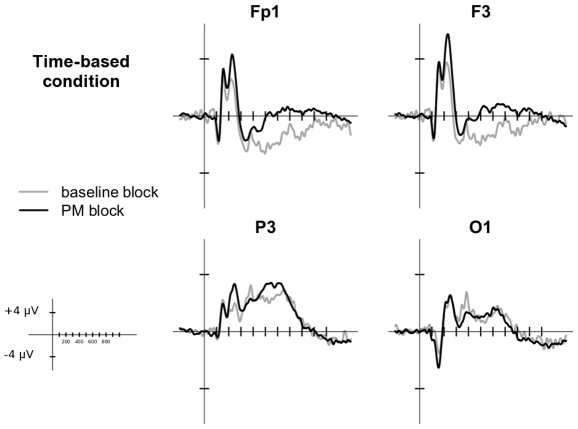
Grand average ERP waveforms for Time-based PM condition. The plots show the ERPs time-locked to ongoing trials in baseline block (gray line) and in PM block (black line) of the electrodes in which the effects were mainly expressed. Since no between-hemisphere differences were found, only left electrodes are reported here.

**Figure 4:**
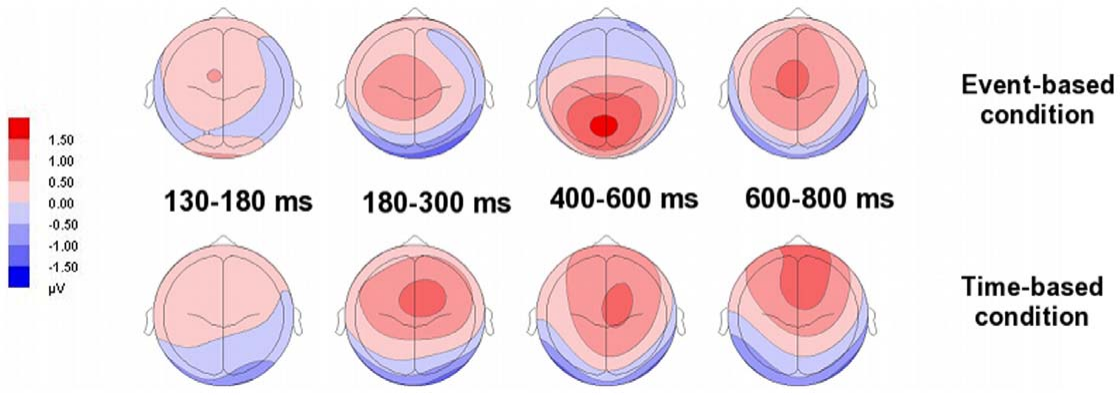
Scalp distribution of ERP differences in Event-based and Time-based PM conditions. The amplitudes shown are obtained as differences PM block-minus-baseline block of the ERPs time-locked to ongoing trials. Average Maps are shown for the time windows in which the ERPs were analysed.

Figure 2 and Figure 3 depict the grand average waveforms of the ERPs in the ongoing trials for event-based and time-based PM conditions in the selected electrodes. ERPs were characterized by an early biphasic modulation with two positive shifts; the first peaking at around 150 ms and the second peaking at around 240 ms. In a subsequent time window (starting at 400 ms), a further positive modulation was observed. In general, in all the time windows investigated, the ERPs in the PM block had widespread more positive amplitudes than the ERPs in the baseline block. The scalp maps in Figure 4 show the differences in the ERP topography between time-based and event-based PM conditions for all the time windows.

### 130–180 ms

In the first time window, the effect of Electrode [F(7, 189) = 11.37, p<0.001, η_G_^2^ = 0.19] was significant. Contrasts exploring the differences among each electrode revealed less positive ERP amplitudes in occipital electrodes (i.e., O1, O2) than in the other electrodes (i.e., Fp1, Fp2, F3, F4, P3, P4; all *ps*<0.01).

More interestingly, the Condition×Block interaction [F(1, 27) = 5.10, p = 0.03, η_G_^2^ = 0.01] was significant. In the event-based PM condition, the mean amplitude was more positive in the trials of the PM block than in trials of the baseline block (*p*<0.05), whereas in the time-based PM condition no differences were observed between the two blocks. This modulation of the early components, shown only in the PM block of the event-based condition, is broadly distributed over the scalp and it could be attributable to the effect of a greater recruitment of attentional resources [57] allocated to the stimulus when a PM cue has to be expected and monitored.

### 180–300 ms

In the second time window, the main effect of Block [F(1, 27) = 11.23, p<0.001, η_G_^2^ = 0.02] was significant, with the ERPs being generally more positive in the PM block than in the baseline block, in both the conditions. Moreover, the Block×Electrode interaction was significant [F(7, 189) = 8.00, p<0.001, η_G_^2^ = 0.01], revealing that such widespread positive modulation was however mainly expressed over frontopolar (Fp1, Fp2) and frontal (F3, F4) electrodes (all *ps*<0.005). This effect seems to indicate that both the time- and the event-based PM tasks might be mediated by frontal activity in order to be carried out.

### 400–600 ms

In this time window, the effect of Electrode [F(7,189) = 16.97, p<0.001, η_G_^2^ = 0.20] was significant, with contrasts indicating that the amplitude was more positive in parietal electrodes than in all the other electrodes (all *ps*<0.001). Waveform, temporal dynamics and distribution over parietal sites reflected those of the P3b, a component traditionally associated with the stimulus evaluation [see 58 for a review].

The effect of Block was significant, with trials in the PM block characterized by a more positive amplitude compared to trials in the baseline block [F(1,27) = 32.46, p<0.001, η_G_^2^ = 0.08]. Finally, the Condition×Block×Electrode interaction was significant [F(7,189) = 4.47, p = 0.02, η_G_^2^ = 0.01]. Post-hoc analysis showed that in frontopolar (Fp1, Fp2) and frontal (F3, F4) electrodes, the amplitude of the ERPs was more positive in PM block compared to the baseline block, in both the event- and the time-based PM conditions (all *ps*<0.05). On the other hand, over occipital and parietal electrodes (P3, P4, O1, O2) only in the event-based PM condition there was a significant difference between baseline and PM blocks, with a greater positivity characterizing trials in the PM block (all *ps*<0.05).

The pattern of results in this time window suggests the presence of two different phenomena: the first is an increased activation in the PM block than in the baseline block, in both the time- and the event-based PM conditions, mainly expressed over frontal sites. The second is an increased parietal and occipital positivity in the PM block only for the event-based PM condition. This latter effect seems to indicate that the P3b component is modulated by the addition of a PM task only in the event-based PM condition.

### 600–800 ms

In the last time window considered, the main effect of Electrode was significant [F(7,189) = 9.13, p<0.001, η_G_^2^ = 0.11]. The contrasts revealed an enhanced positivity over parietal electrodes as compared to all the other electrodes (all *ps*<0.005).

The effect of Block [F(1,27) = 9.69, p<0.001, η_G_^2^ = 0.04] was significant, with a more positive amplitude in the PM than in the baseline Block, as well as the Block×Electrode interaction [F(7,189) = 10.95, p<0.001, η_G_^2^ = 0.03]. Post-hoc analysis exploring the interaction revealed that, as compared to the baseline block, trials in the PM block showed a more positive amplitude over frontopolar and frontal electrodes (all *ps*<0.005), regardless of the PM condition. These findings reproduce the ERP pattern over frontal and frontopolar electrodes also evident in the 180–300 ms and the 400–600 ms windows. This consistent pattern suggests a sustained frontal activity (common for time-based and event-based PM conditions) in blocks that require performing a PM task.

## Discussion

The present study provides the first evidence of commonalities and differences in the electrophysiological correlates of Strategic Monitoring between time-based and event-based prospective memory tasks. In general, the addition of a time-based or an event-based PM task to an ongoing activity led to a similar sustained and positive modulation of the ERPs in the ongoing trials, broadly distributed but particularly expressed over prefrontal and frontal regions. On the other hand, two further ERP modulations were selectively found in the event-based PM task: an increased positivity over occipital and parietal regions occurring between 400–600 ms post-stimulus, and an early modulation, occurring between 130–180 ms post-stimulus. The meaning of such modulations will be discussed in the light of the theoretical framework by Guynn [6], [10], which described Strategic Monitoring as being composed of two mechanisms: retrieval mode and target checking.

Specifically, in order to compare Strategic Monitoring in time-based and event-based PM tasks, the behavioural performance and the ERPs elicited by the ongoing trials were analysed in the two PM conditions. The RTs and the ERPs relative to the block in which participants performed merely the ongoing task (baseline block) were compared with those in which participants were required to concurrently perform the PM task (PM block). Since the participants belonging to the two conditions performed a different PM task (time-based or event-based) but the same ongoing task, possible RTs and ERP differences evident in the PM block should be interpreted as reflecting different mechanisms supporting the two PM tasks.

Concerning the behavioural results, the involvement of Strategic Monitoring was reflected in both the PM conditions by the slowing down of the RTs in the PM block as compared to the baseline block, after removing statistically the practice effect.

The investigation of ERPs allowed us to better clarify which Strategic Monitoring mechanism was common in time-based and event-based tasks and which was specific to the event-based PM task. Consistently with the previous electrophysiological studies of event-based PM [34]–[39], also in our event-based condition, the addition of a PM instruction to the ongoing task led to a sustained increased and widespread ERP activity, relative to the ERPs elicited by the ongoing trials in the baseline block. Interestingly, a similar pattern of sustained ERP activity was found when individuals had to accomplish a time-based PM task. Particularly, the two PM tasks shared an increased positivity starting at 180 ms post-stimulus and lasting until 800 ms, broadly distributed over the scalp, but mostly expressed over frontal and prefrontal sites. Importantly, these results suggest that, although in the time-based task the processing of ongoing stimuli was irrelevant for executing the prospective intention, the ERPs elicited by these stimuli were, however, modulated by Strategic Monitoring. This common ERP activity might reflect a mechanism of Strategic Monitoring that is equally engaged in time- and event-based PM tasks, namely the retrieval mode [6], [10]. Indeed, in both the time-based and the event-based PM tasks, the prospective intention has to be maintained active in mind across the ongoing trials, in preparation for executing the intended action. Moreover, the frontal and prefrontal distribution of these ERP modulations is in line with the findings from the other ERP studies [34], [35], [38] and might provide support for the notion that the retrieval mode is mediated by the activity in the frontal cortex [43], [44], [59]–[66]. It might also extend the results of the neuroimaging studies suggesting that prefrontal cortex is implied in maintaining delayed intentions regardless of their nature, being active during the maintenance not only of the event-based PM intentions [24], [25], [30] but also of the time-based ones [31], [67]. Furthermore, it is possible that this frontal activity reflected the engagement of executive resources required for managing and holding in mind more tasks/goals simultaneously [68]. Nevertheless, even if multi-task managing could have contributed to determine this frontal activity, it is unlikely to suppose that this was the only process related to the ERP modulations. Indeed, the ongoing task adopted was low demanding, and this choice was made in order to reduce the cost of managing more tasks concurrently. Moreover, we cannot exclude the influence of another process that is specifically involved in time-based PM, namely the internal time estimation. Although we tried to reduce the engagement of the time estimation allowing participants to check the clock whenever they needed, it is not possible to completely exclude the influence of this operation on the ERPs. Thus, an interesting aim of future studies could be that of investigating the influence of time estimation on the ERPs elicited by ongoing activity, for example imposing some limitations in the use of the clock.

Together with similarities, we also found differences in the ERP modulations between the two PM tasks. Specifically, the ERPs in the PM block of the event-based PM condition were characterized by an enhanced positivity between 400 and 600 ms post-stimulus relative to the ERPs in the baseline block, over parietal and occipital regions. Such posterior ERP modulation was not revealed in the PM block of the time-based PM condition. This different pattern of modulations most likely reflected the different type of target checking engaged for the two PM tasks [6], [10]. The difference in the ERP modulation is explained by the fact that, in the event-based PM task, additional attentional resources are allocated to the incoming stimulus for assessing whether its features match with those of the event-based PM cue [6], [12]. These additional resources are not required in the time-based PM task, where target checking does not operate by monitoring the ongoing stimuli but rather by checking the clock. The idea that such increased positivity in the event-based PM task is the ERP correlate of target checking is also driven by the fact that it occurred in the time window corresponding to the stage of stimulus processing. Indeed it seemed to be a modulation of the P3b, which is an index of stimulus evaluation sensitive to the degree of attention required to elaborate the stimulus [see 58 for an updated review]. This result confirms the claim that target checking is mediated by the allocation of increased attentional resources to the stimulus [11]. Finally, the ERP modulation was distributed mainly over occipital-parietal regions, which were shown to play a crucial role in the detection of the PM cue [69]–[71].

Another difference between the ERPs in the ongoing trials of the time-based and the event-based PM condition was found in the modulation of an earlier ERP component. As compared to the baseline, the trials in the PM block of the event-based, but not of the time-based condition elicited a broadly distributed increased amplitude of the phasic component occurring between 130–180 ms post-stimulus. This ERP modulation seems unlikely to be explained by target checking, since it occurred very early. Rather, it might presumably reflect the recruitment of attentional resources required to be in a state of readiness and preparedness, in order to later recognize and respond to the ongoing stimuli as a PM cue. This state of readiness to respond would differ in the two PM tasks, presumably because the occurrence of the PM cue is unpredictable in event-based PM tasks, but not in time-based ones. Thus, a greater level of attentional resources devoted to such process should be required in event-based than in time-based PM tasks. A similar early modulation was shown in another study concerning the event-based PM [36], in which an enhanced positivity was shown at 140 ms post-stimulus over the occipital-parietal regions. Likewise to our interpretation, they suggested that such modulation was the expression of the preparatory attention which, supporting early visual processing, could then facilitate target checking.

The concept of a readiness to respond has been incorporated in the concept of the retrieval mode in Guynn's model [6], [10]; nevertheless our findings might suggest the presence of two separate aspects of retrieval mode, differentially involved in the time-based and the event-based PM tasks. Specifically, time-based and event-based PM seem to share the retrieval mode proper, conceptualised as maintaining active the prospective intention in memory, and evident in our experiment as a sustained frontal activity. Whereas another mechanism, that we call “readiness mode”, distinguishes the two PM tasks, with the event-based PM task involving higher attentional preparatory resources for this state, relative to the time-based PM task. The readiness mode can be better described as an attentional sustained condition for being prepared to process the incoming stimuli as possible PM cues. The distinction between a readiness mode and a retrieval mode is in line with Smith's model, which differentiates preparatory attentional processes and memory processes, required for monitoring the presence of an event PM cue [11].

Summarizing, our study showed commonalities and differences in the ERP modulations of Strategic Monitoring in event- and time-based PM tasks. A similar frontal sustained activity characterized the ongoing trials of both the PM tasks, and it seems to reflect the memory mechanism of holding the prospective intention in mind, i.e. the retrieval mode. This is an important finding since, to our knowledge, this is the first evidence of the electrophysiological correlate of such Strategic Monitoring mechanism in the time-based PM. In addition, the comparison between a time-based PM task and an event-based PM task allowed us to better clarify the ERP indices of the different mechanisms implied in Strategic Monitoring in the event-based PM. The enhanced positivity over occipital and parietal regions at 400 ms post-stimulus in the event-based condition seems to reflect the increased recruitment of resources required for checking the occurrence PM cue, whereas the early modulation, broadly distributed over the scalp, could be the expression of the allocation of attention for being in a readiness mode to execute the PM response. Finally, our findings suggest a reviewed classification of the sustained mechanisms mediating Strategic Monitoring, by distinguishing between a “retrieval mode”, more related to memory processes, and a “readiness mode”, more related to attentional processes.

## Supporting Information

File S1 **Mixed effect regression analysis for the investigation of the PM interference effect.**
(DOC)
