# Supplementary material for: Electrophysiological Correlates of Strategic Monitoring in Event-Based and Time-Based Prospective Memory
Source: PLoS One. 2012 Feb 21;7(2):e31659. doi: 10.1371/journal.pone.0031659 (PMC3283681; doi:10.1371/journal.pone.0031659)
Supplement: File S1 — Mixed effect regression analysis for the investigation of the PM interference effect. (DOC) [file pone.0031659.s001.doc]

**FILE S1 – Mixed effect regression analysis** **for the investigation of the PM interference effect**

Since in the present study the PM block always followed the baseline block, a PM interference effect (i.e., decline of ongoing performance after receiving PM instructions) could have been masked by the concurrent speeding associated with practice effect. In order to disentangle between these two effects, we run a further analysis inserting the trial number as a covariate. In this procedure each trial is associated with a progressive number starting from 1 (first trial of the baseline block) to 40 (last trial of the 40 trials of the baseline block) and then from 41 (first trial of the PM block) to 390 (last trial of the 350 trials of the PM block). In this way, each trial is associated with the ordinal number indicating its position within the whole experiment (the 1st trial, the 2nd trial, and so on) regardless of the block it belongs to. By inserting the trial as a covariate it is possible to investigate the effect of Block, once the effect of trial (i.e. the practice effect) is partialled out. We performed this analysis by means of mixed effect regression modelling [1,2]. Mixed model regressions (henceforth, mixed models), as the multiple regressions, allow to study the effect of both factors and covariates. As additional value, they allow to deal with both fixed effects controlled by the experimenter (e.g., the condition and the block type in the present experiment) and random effects, i.e. factors whose levels are randomly sampled from a population (e.g., the participants which took part to the experiment). When taking into account both fixed and random effects, the whole structure of data is considered, thus leading to enhanced statistical power. We chose mixed models for three main reasons: 1) they warranted us the best statistical power available; 2) they prevented us from splitting arbitrarily the PM block in different sub-blocks (e.g., first sub-block of 40 trials, second sub-block of 40 trials and so on) to study the effect of trial number, allowing to keep the variable as continuous [3]; and 3) they allowed to consider each participant separately, whereas in a traditional regression, data from all participants would have been averaged. Data utilized to fit the model were the RTs associated with each single response of each single participant. Mixed models permit indeed to include all of the observations collected (and not the responses averaged by subject), still protecting against inflated significance. For the mixed effect model we utilized the lme4 R package [4]. The mixed model fitted on data had the following initial structure: one dependent variable, three variables included as fixed effects and three variables included as random effects. RTs were included as a dependent variable. Before being entered in the analysis they were log-transformed in order to reduce data skewness. The four fixed effects considered were: Condition as a two level factor (Condition=TIME, Condition=EVENT), Block as a two level factor (Block=baseline, Block=PM), Response required as a two levels factor (Response=same, Response=different), and Trial number, included as a covariate. All factors were codified as dummy variables before entered in the analysis. Trial number was included as a continuous covariate as follows: trials belonging to the baseline block (n = 40) were associated with increasing numbers from 1 to 40. Trials belonging to the PM block (n = 350) were associated with increasing numbers starting from 41. The random effects considered in the model were the effect of Participants, the effect of Block nested within participants, and the Random slope for trial number adjusted for participants. By considering these variables as random effects, some sources of variance of data were taken into account, thus improving the statistical power of the analysis. Starting from an initial model, which included all the variables listed above, the model which best fitted the data (henceforth, the final model) was selected through a backfitting procedure: non-significant variables were excluded from the model one at a time, starting with the variable with the lowest | t |. For factors, no effect was excluded if it belonged to a factor in which at least one level had a | t | > 2. Before excluding a variable from the model, a further check was made: a likelihood ratio test was carried out and only if the presence of the variable was irrelevant to improving the goodness-of-fit of the model, was the variable definitively removed. Following this procedure, the final model identified included all initial variables except Condition, which was removed from the model. The non-significance of this variable indicates that no differences in predictions are expected if the condition is time-based or event-based.

Briefly, the mixed model analysis yielded the following results: RTs were slower in the PM block than in the baseline block, slower when the required response was “different” than when it was “same”, and faster as the trial ordinal position increased (confirming a significant practice effect). Detailed results of the analysis are listed in Table S1 and are interpreted as follows: the Intercept, (6.66) is the default prediction for stimuli when the response is “same” in the baseline block (response=different, block=baseline). For the PM block (Block=PM) the expected modification in the Intercept is +0.058, so the expected RTs are higher compared to the default prediction, when block=baseline. When the response is “same” (response= same) the expected modification to the Intercept is -0.060, so faster compared to the expected RTs when response=different. The effect of Trial number is associated with the coefficient β = - 0.0002[[1]](#footnote-2), that in the case of a covariate indicate a slope. The influence of every fixed effect is calculated partialling out the influence of the other significant fixed effects. Importantly to our investigation, this means that the increase of RTs in the PM block compared to the baseline block is significant after partialling out the effect of Trial number (i.e. the practice effect). The final model included also three random effect parameters: the effect of Participants, the effect of Block nested within participants and the Random slope for trial number adjusted for participants. The significance of these effects indicates that taking into account these sources of error improves the goodness of fit of the model. The effect of Participants indicates that there is a significant variability in the overall performance of every participant. The effect of Block nested within participants indicates that there is a significant variability across participants in the effect of block, i.e. some participants show a bigger PM interference effect than others. The effect of Random slope for trial number adjusted for participants indicates that there is a significant variability in the slope of the covariate Trial number, that is, participants show different degrees of practice effect. Fixed effects are listed in Table S1 and random effects in Table S2. Summarizing, mixed effects model showed the otherwise masked PM interference effect: the PM block had significantly higher RTs compared to the baseline block in both event-based and time-based conditions, when controlling for the practice effect and achieving enough statistical power.

**Table S1. Fixed effects of mixed model analysis.**

| **Parameters** | **β (SE)** | **t** | ***p* value^2^** |
| --- | --- | --- | --- |
| Intercept  *Block* =baseline  *Response* =different” | 6.61 (0.026) | 256.80 | < 0.001 |
| *Block* = PM | 0.058 (0.020) | 2.96 | < 0.005 |
| *Response* = “same” | -0.060 (0.003) | -17.00 | < 0.001 |
| Trial number | -0.0002 (0.00001) | -13.74 | < 0.001 |

^2^ *p* values were calculated through Markov Chain Monte Carlo sampling (10'000 samples), as implemented in the function *pvals.fnc* of *languageR* package [2,5].

**Table S2. Random effects of mixed model analysis**

| **Parameters** | **SD** |
| --- | --- |
| Participants | 0.07 |
| Block nested within participants | 0.12 |
| Random slope for trial number adjusted for participants | 0.00021 |

**References**

1. Pinheiro JC, Bates DM (2000) Mixed-Effects Models in S and S-PLUS. New York: Springer.
2. Baayen RH, Davidson DJ, Bates DM (2008) Mixed-effects modelling with crossed random effects for subjects and items. J Mem Lang 59: 390–412.
3. Cohen J (1983) The Cost of Dichotomization. Appl Psych Meas 7: 249-254.
4. Bates D, Maechler M, Bolker B (2011) lme4: Linear mixed-effects models using S4 classes. R package version 0.999375-42. Available: <http://CRAN.R-project.org/package=lme4>. Accessed 2011 Dec 22.
5. Baayen, R. H. (2011). languageR: Data sets and functions with "Analyzing Linguistic Data: A practical introduction to statistics". R package version 1.2. Available: <http://cran.r-project.org/web/packages/languageR/index.html>. Accessed 2011 Dec 22.

1. As in traditional multiple regression, positive coefficients indicate an increase in RTs whereas negative coefficients indicate a decrease in RTs, coefficients associated with factors are to be interpreted as adjustment to Intercept, whereas coefficients associated with continuous variables are to be interpreted as adjustment to the slope. [↑](#footnote-ref-2)
